# Discovery of a Novel Variant of SEMA3A in a Chinese Patient with Isolated Hypogonadotropic Hypogonadism

**DOI:** 10.1155/2021/7752526

**Published:** 2021-10-21

**Authors:** Wenting Dai, Jia-Da Li, Xinying Wang, Wang Zeng, Fang Jiang, Ruizhi Zheng

**Affiliations:** ^1^Department of Clinical Laboratory, The Affiliated Zhuzhou Hospital Xiangya Medical College, Central South University, Zhuzhou, Hunan 412007, China; ^2^School of Life Sciences, Central South University, Changsha, Hunan 410078, China; ^3^Hunan Key Laboratory of Animal Models for Human Diseases, Central South University, Changsha, Hunan 410078, China; ^4^Hunan Key Laboratory of Medical Genetics, Central South University, Changsha, Hunan 410078, China; ^5^Department of Endocrinology, The People's Hospital of Henan Province, Zhengzhou, Henan 450003, China

## Abstract

Semaphorin (SEMA) has an important role in nerve development, organ formation, immune response, angiogenesis, and tumor growth. SEMA can regulate the growth and branching of axons, the morphology of dendrites, and the migration of neurons. The loss-of-function in SEMA and its receptors PLXNs and NRP affect the migration of GnRH neurons, leading to idiopathic hypogonadotropic hypogonadism (IHH). As a member of the SEMA family, SEMA3A has an important role in axonal rejection, dendritic branching, synaptic formation, and neuronal migration. There are more and more SEMA3A variants identified in IHH patients. In this study, we identified a novel SEMA3A variant (c.1369A > G (p.T457A)) in a male nIHH patient. Functional studies indicated that the T457A SEMA3A variant led to the defect of FAK phosphorylation and GN11 cell migration, which strongly argued in favor of its pathogenic effect in the nIHH patient. Our findings substantiated that the 435–457 position of SEMA3A might be very important for the secretion of SEMA3A. Haploin-sufficiency of SEMA3A in humans was sufficient to cause the IHH phenotype. SEMA3A variants might have a role in modifying the IHH phenotype, according to the variants at different positions of SEMA3A. SEMAs and its receptors formed a complex network, and other members of the SEMA-signaling pathway might also be involved in the pathogenesis of IHH.

## 1. Introduction

SEMA is a family of proteins widely involved in various physiological and pathological processes, such as nerve development, organ formation, immune response, angiogenesis, and tumor growth. According to its structural characteristics, SEMA family can be divided into 8 subtypes [1]. The specific receptors of SEMA family mainly include plexin (PLXN) and neuropilin (NRP). During the development of the nervous system, SEMA can regulate the growth and branching of axons, the morphology of dendrites, and the migration of neurons. Recent studies have found that some mutations in SEMA and its receptors PLXNs and NRP affect the migration of GnRH neurons, resulting in delayed puberty maturation or infertility [2], leading to idiopathic hypogonadotropic hypogonadism (IHH).

Mutations in SEMA3A were recently identified in IHH patients [3–6]. SEMA3A, as a key signal protein in axonal development, participates in many physiological processes. It is a secretory protein that participates in axonal rejection, dendritic branching, synaptic formation, and neuronal migration by binding to NRP1, NRP2, and PLXNA complex receptors [7, 8].

In this study, we identified a novel SEMA3A variant in a large cohort of IHH patients from China. Functional studies suggested that the SEMA3A mutation led to the defect of FAK phosphorylation and GN11 cell migration.

## 2. Materials and Methods

### 2.1. Patients

196 unrelated IHH patients (161 males and 35 females, 126 with KS and 70 with nIHH) were gathered among the Xiangya Hospital (Changsha, China) and People's Hospital of Henan Province (Zhengzhou, China). The patients or their adult parents signed an informed consent form. The studies were approved by the Ethics Committee of School of Life Sciences, Central South University (No. 2017030801). Pedigree analysis was studied if available.

### 2.2. Whole Exome Sequencing

The exome depth algorithm was used for analyzing copy number variation (CNV) [9]. The detected variants were verified by PCR-Sanger sequencing, and the sequences of PCR primers are given in Table S1. Cosegregation analyses were conducted on all family members if available.

The pathogenicity prediction of the identified variants is described previously [3]. The Genome Aggregation Consortium (gnomAD) and the Chinese control individuals were used to determine the allelic frequencies for each variant. In addition, we used InterVar (http://wintervar.wglab.org/) to determine variant classification based on American College of Medical Genetics and Genomics (ACMG) guidelines [10].

### 2.3. Antibodies and Reagents

Antibodies against SEMA3A (ab18554), PAK (ab30931), and pFAK (ab3350) were purchased from Abcam.

### 2.4. Plasmids and Cell Transfection

The pRK5-SEMA3A-Flag constructs (SEMA3A cDNA, GenBank accession no. NM_006080) were constructed at Youbao Biological Company. We used the QuikChange mutagenesis kit (Stratagene, La Jolla, CA) to make variants in the pRK5-SEMA3A-Flag constructs by site-directed mutagenesis. All constructs were verified by nucleotide sequencing.

Plasmid transfections were performed with Lipofectamine 2000 (Invitrogen, Carlsbad, CA) reagents according to the manufacturer's protocol.

### 2.5. Expression and Secretion of SEMA3A Protein

We transfected pRK5-SEMA3A-Flag and T457A mutant plasmid in the HEK293T cells through Lipofectamine 2000 reagent (Invitrogen, Carlsbad, CA). We used the Ultra 3K filter tubes (30 kDa NMWL, Millipore) to concentrate the media of the HEK293T cells. Western blot was used to detect the expression and secretion of SEMA3A protein. Each experiment was performed at least three times.

### 2.6. Detection of FAK Phosphorylation in GN11 Cells

GN11 cells maintained in the serum-free medium were stimulated with WT and mutant SEMA3A concentrated conditioned media for 20 minutes. Western blot was used to detect the expression of pFAK and FAK in cell lysates. Each experiment was performed at least three times.

### 2.7. Transwell Assay

GN11 cells (1 × 10^4^ cells) planked into transwell chambers with 8 *μ*m pore filters (Sigma, WA) were maintained in SEMA3A and its mutant conditioned media overnight. The cells removed mechanically from the upper side of the filters were stained by DAPI and counted through the fluorescence microscope.

### 2.8. Statistical Analysis

Statistical analysis was performed by the Prism 6.01 (GraphPad Software, San Diego, CA) with the unpaired two-tail Student's *t*-test or a repeated-measure ANOVA followed by Bonferroni post hoc tests to analyze the data for differences. *P* < 0.05 was considered significant. The distribution of data points is shown as mean ± S.E.M.

## 3. Results

### 3.1. Identification of a Novel SEMA3A Variant in Patients with IHH

**Figure 1:**
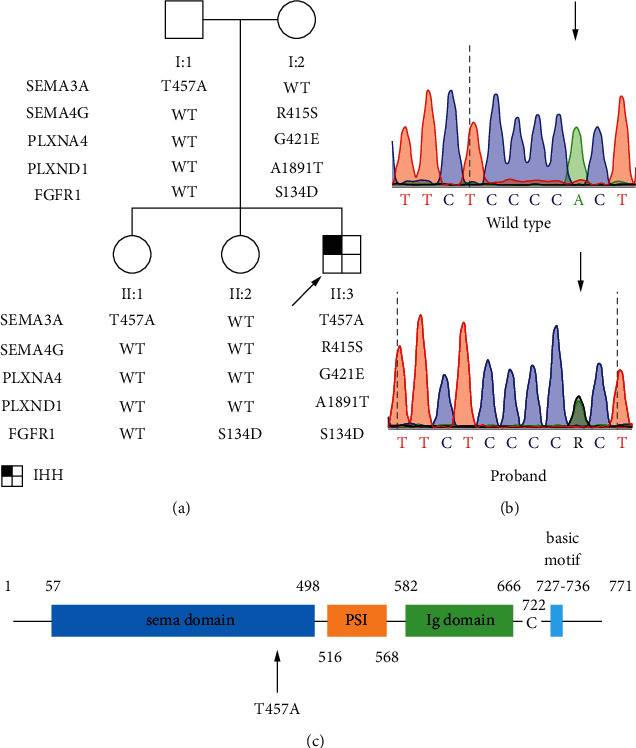
A novel SEMA3A variant screened in IHH patients. (a) Pedigree analysis of the proband. The proband is indicated by a black arrow. Circles and squares suggest women and men, respectively. (b) Sequence analysis of WT (top) and SEMA3A mutation (bottom). The arrow above the chromatogram indicates the codon affected by the variant. (c) The location of the T457A variant. SEMA, semaphorin domain; PSI, plexin/semaphorin/integrin domain; Ig, immunoglobulin-like domain; C, cysteine residue; IHH, idiopathic hypogonadotropic hypogonadism.

We screened a SEMA3A heterozygous variant (c.1369A > G (p.T457A)) in a male proband of nIHH (Figure 1(b)). The T457A variant was located in the SEMA domain (Figure 1(c)).

Clinical data of the proband are given in Table 1. Interestingly, the proband (II : 3), carrying p.T457A SEMA3A, p. R415S SEMA4G, p. G421E PLXNA4, p. A1891T PLXND1, and p.S134D FGFR1 variants, was diagnosed as nIHH. His mother (I : 2) carrying p.R415S SEMA4G, p.G421E PLXNA4, p.A1891T PLXND1, and p.S134D FGFR1variants and his father (I : 1) carrying p.T475A SEMA3A variant have normal puberty and fertility. His sister (II : 1) with p.T475A SEMA3A variant and his sister (II : 2) with p.S134D FGFR1 variant showed normal puberty and fertility (Figure 1(a)).

### 3.2. Functional Analysis of Novel SEMA3A Variant

**Figure 2:**
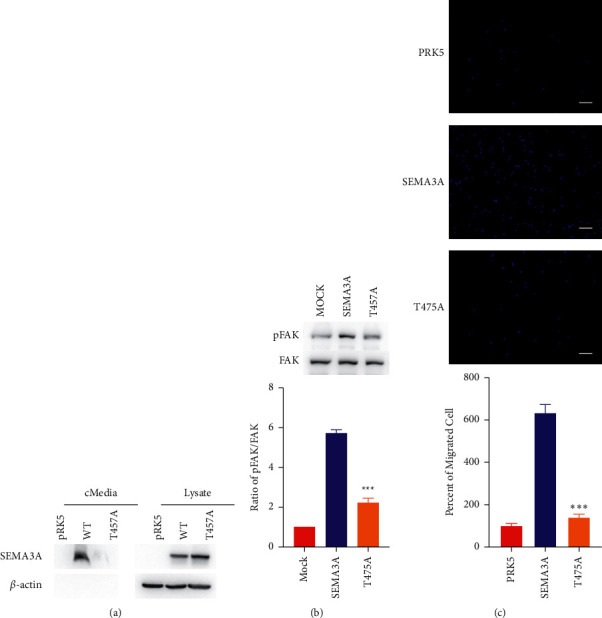
Functional study of the novel SEMA3A variant. (a) Expression and secretion of WT SEMA3A protein and T457A mutant protein. (b) Above: GN11 cells incubated with the serum-free medium (mock, negative control) or conditioned medium containing WT and mutated SEMA3A protein for 20 minutes. The phosphorylated FAK (pFAK) and total FAK in GN11 cells detected by Western blot. Below: the bar chart shows the ratio of pFAK to total FAK. ^∗∗∗^*P* < 0.005. (c) Above: transwell assay of GN11 cells incubated with the serum-free medium (pRK5, negative control) or the conditioned medium containing WT and mutated SEMA3A protein. Below: the statistic analysis of the transwell assay ^∗∗∗^*P* < 0.005. Scale bars: 30 mm.

Through Western blot analysis, both wild-type (WT) SEMA3A protein and T457A mutant protein were expressed in HEK293T cells. But, the T457A mutant protein was defective in secretion. Then, we added the conditioned medium containing concentrated WT and T457A mutant SEMA3A protein to GN11 cells and detected the phosphorylation level of FAK (pFAK) in GN11 cells by Western blot. We found that pFAK in GN11 cells stimulated by WT SEMA3A protein was significantly increased compared with that in the negative control, but the T457A mutant protein could not effectively induce FAK phosphorylation in GN11 cells (Figure 2(b)).

Furthermore, we detected the migration of GN11 cells maintained in WT SEMA3A and T457A mutant protein by the transwell assay. Compared with WT SEMA3A protein, the migration of GN11 cells was significantly decreased under the stimulation of T457A mutant protein (Figure 2(c)). It suggested that the T475A variant resulted in the loss of SEMA3A function.

## 4. Discussion

The development of the mammalian reproductive system depends on GnRH neurons located in the hypothalamus. GnRH neurons first appear in the medial olfactory placode and migrate to the cribriform plate and forebrain along the axons of olfactory vomeronasal neurons. Then, GnRH neurons enter the forebrain, migrate to the developing olfactory bulb, and finally reach the ventral hypothalamus. As a neuroendocrine cell, they release GnRH into the pituitary portal circulation [11] and regulate the “hypothalamus pituitary gonad (HPG) axis” of human beings. Accordingly, loss-of-function for molecules, such as SEMA3A that control olfactory or vomeronasal axon-patterning perturbs GnRH neuron migration, leads to delayed or absence of pubertal maturation and infertility [2]. The normal secretion of SEMA3A plays an important role in the normal function of SEMA3A.

**Figure 3:**
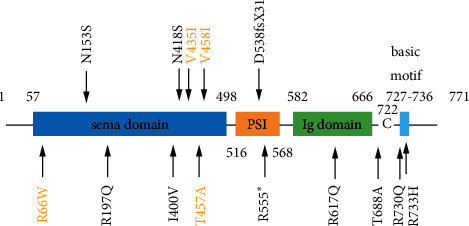
Schematic representation of the SEMA3A protein. Location of the SEMA3A variants characterized in IHH. Four variants caused impaired secretion of SEMA3A highlighted in yellow. SEMA, semaphorin domain; PSI, plexin/semaphorin/integrin domain; Ig, immunoglobulin-like domain; C, cysteine residue.

In this study, we further investigated 196 Chinese IHH patients and found a novel SEMA3A variant (p.T457A) in a male nIHH patient. Functional study suggested that it led to loss-of-function in SEMA3A, and it resulted in impaired secretion of SEMA3A. Dai et al. found three SEMA3A variants (R197Q, R617Q, and V458I) in 177 Chinese IHH patients by whole exome sequencing. Functional study suggested that all three variants caused loss-of-function in SEMA3A [3], and the V458I variant led to impaired secretion of SEMA3A. Hanchate et al. identified a frameshifting small deletion (D538fsX31) and several heterozygous missense variants (D538fsX31, R66W, V435I, N153S, I400V, T688A, and R733H) in SEMA3A in KS patients. Among them, three variants resulted in impaired secretion of SEMA3A (D538fsX31, R66W, and V435I) [4]. In conclusion, the R66W, N153S, R197Q, I400V, V435I, p.T457A, and V458I variants are located in SEMA domain, all in heterozygosity, but the R66W, V435I, V458I, and p.T457A variants caused impaired secretion of SEMA3A (Figure 3). It suggested the mutations at positions 435–457 in the SEMA region are likely to lead to impaired secretion of SEMA3A, and the 435–457 position is very important for the secretion of SEMA3A. Haploin-sufficiency of SEMA3A in humans was sufficient to cause the IHH phenotype [3, 4, 6, 12].

SEMA3A was considered to play an important role in axonal rejection, dendritic branching, synaptic formation, and neuronal migration [8]. SEMA3A knock-out mice exhibited abnormal olfactory bulb innervation and altered development of GnRH neurons [7]. Hanchate et al. found several variants in SEMA3A in KS patients [4]. Young et al. found a heterozygous deletion of 213 kb at locus 7q21.11 in a family, and the family members with KS had no other clinical neurological abnormalities [6]. Kansakoski screened three heterozygous variants (N153S, N418S, and V435I) in SEMA3A in three KS patients [5]. Dai et al. found that two out of three patients with SEMA3A variants (R197Q and R617Q) showed optic nerve defects [3]. Interestingly, the patient with SEMA3A variant (T457A) had neither visual impairment nor olfactory defect. Gileta et al. screened a nonsense variant (R555^∗^) and a deletion of exons 15, 16, and 17 in SEMA3A in a female KS patient [12]. It seems possible that SEMA3A variants might modify the IHH phenotype, according to the variants at different positions of SEMA3A.

Furthermore, SEMAs and its receptors form a complex network. Some SEMAs can bind to a variety of receptors. On the other hand, some SEMA receptors can bind to many kinds of SEMAs [13]. In this study, we found the proband (II : 3) carrying T457A SEMA3A, R415S SEMA4G, G421E PLXNA4, A1891T PLXND1, and S134D FGFR1 variants. Giacobini et al. reported that SEMA4D and plexin B1 are important for the guidance of migrating GnRH neurons [14]. It suggested that other members of the SEMA-signaling pathway might also be involved in the pathogenesis of IHH.

Notably, Hanchate et al. found that the patients carrying the SEMA3A variants (T688A, I400V, and V435I) also carry KAL1 variant (Y217D), PROKR2 variant (R268C), PROK2 variant (H70fsX5), and FGFR1 variant (G687N), respectively [4]. Laitinen et al. found that two patients with SEMA3A variants (N153S and N418S) also had truncating mutations in FGFR1 [15]. Alkelai et al. discovered rare heterozygous FGFR1 and SEMA3A variants in one IHH families [16]. Dai et al. discovered that one proband with SEMA3A variant (R617Q) also had p.Q256K and p.R270H variants in CCDC141 and PROKR2 [3]. In this study, the proband with the SEMA3A variant (T457A) also carry p.S134D variant in FGFR1. It suggested that the SEMA3A-NRP1/NRP2-PLXNA1 signaling pathway might cooperate with the PROK2-PROKR2 or FGF8-FGFR1 signaling pathway in the pathogenesis of IHH.

In summary, we present a new SEMA3A variant identified in 196 Chinese IHH patients. From our results, we concluded that the p.T457A variant affected the secretion of SEMA3A and, furthermore, influenced signaling activity of SEMA3A, which led to its pathogenic effect in the nIHH patient. Variants at positions 435–457 in the SEMA region was likely to lead to impaired secretion of SEMA3A, and the 435–457 position might be very important for the secretion of SEMA3A. Furthermore, SEMAs and its receptors form a complex network, and other members of the SEMA-signaling pathway might also be involved in the pathogenesis of IHH. Otherwise, it will be worthful to study whether the SEMA3A-NRP1/NRP2-PLXNA1 signaling pathway cooperated with the PROK2-PROKR2 or FGF8-FGFR1 signaling pathway in the pathogenesis of IHH.

## Figures and Tables

**Table 1:** Clinical data of the IHH proband carrying SEMA3A variant.

ID	Dx	c.HGVS	p.HGVS	Hormone levels				Other phenotypes	Segregation	MAF		ACMG	In silico prediction			
				LH	FSH	T	E			Matched	All		CADD	SIFT	PolyPhen2	MutationTaster
1	nIHH	SEMA3A c.1369G > A	p.R457Q	0.09	0.25	1	19	—	Pat	0	0	U	25.5	T	P	D
		SEMA4G:c.1243C > A	p.R415S						Mat	0.009	0.0007	U	22.7	T	P	D
		PLXNA4:c.1262G > A	p.G421E						Mat	0.001	0.0001	U	23.6	D	P	D
		PLXND1:c.5671G > A	p.A1891T						Mat	0.004	0.0003	U	25.4	T	P	D
		FGFR1 c.400_401delinsGA	p.S134D						Mat	0	0	LP	22.8	D	P	D

Normal adult men reference range for testosterone (T) 1.75–7.81 ng/mL, for estradiol (E2) < 53 pg/mL, for luteinizing hormone (LH) 1.2–8.6 mIU/mL, and for follicle-stimulating hormone (FSH) 1.3–19.3 mIU/mL. Dx, diagnosis; KS, Kallmann syndrome; nIHH, normosmic idiopathic hypogonadotropic hypogonadism; HGVS, Human Genome Variation Society; Mat, maternal. Pat, paternal. MAF, minor allele frequency in gnomAD database; Matched, ethnically matched population in gnomAD; All, all populations in gnomAD. ACMG criteria: P, pathogenic; B, benign; U, uncertain significance; LP, likely pathogenic; LB, likely benign. CADD, combined annotation-dependent depletion. CADD, SIFT, PolyPhen2, and MutationTaster (MutationT) were used for in silico prediction for the effect of the variants. SIFT: T, tolerated; D, deleterious. PolyPhen2: B, benign; P, possibly damaging; D, probably damaging. MutationTaster: N, polymorphism; D, disease causing.

## Data Availability

The data generated during the study are available from the corresponding author upon request.

## Supplementary Materials

Supplementary Materials Table S1: the sequences for PCR primers.

## References

[1] Tamagnone L., Comoglio P. M. (2000). Signalling by semaphorin receptors: cell guidance and beyond. *Trends in Cell Biology*.

[2] Cariboni A., Hickok J., Rakic S. (2007). Neuropilins and their ligands are important in the migration of gonadotropin-releasing hormone neurons. *Journal of Neuroscience*.

[3] Dai W., Li J. D., Zhao Y. (2020). Functional analysis of SEMA3A variants identified in Chinese patients with isolated hypogonadotropic hypogonadism. *Clinical Genetics*.

[4] Hanchate N. K., Giacobini P., Lhuillier P. (2012). SEMA3A, a gene involved in axonal pathfinding, is mutated in patients with Kallmann syndrome. *PLoS Genetics*.

[5] Känsäkoski J., Fagerholm R., Laitinen E.-M. (2014). Mutation screening of SEMA3A and SEMA7A in patients with congenital hypogonadotropic hypogonadism. *Pediatric Research*.

[6] Young J., Metay C., Bouligand J. (2012). SEMA3A deletion in a family with Kallmann syndrome validates the role of semaphorin 3A in human puberty and olfactory system development. *Human Reproduction*.

[7] Cariboni A., Davidson K., Rakic S., Maggi R., Parnavelas J. G., Ruhrberg C. (2011). Defective gonadotropin-releasing hormone neuron migration in mice lacking SEMA3A signalling through NRP1 and NRP2: implications for the aetiology of hypogonadotropic hypogonadism. *Human Molecular Genetics*.

[8] Alto L. T., Terman J. R. (2017). Semaphorins and their signaling mechanisms. *Methods in Molecular Biology*.

[9] Men M., Li W., Chen H. (2020). Identification of a novel CNV at 8q13 in a family with branchio‐oto‐renal syndrome and epilepsy. *The Laryngoscope*.

[10] Richards S., Aziz N., Aziz N. (2015). Standards and guidelines for the interpretation of sequence variants: a joint consensus recommendation of the American College of medical genetics and Genomics and the association for molecular pathology. *Genetics in Medicine*.

[11] Wierman M. E., Kiseljak-Vassiliades K., Tobet S. (2011). Gonadotropin-releasing hormone (GnRH) neuron migration: initiation, maintenance and cessation as critical steps to ensure normal reproductive function. *Frontiers in Neuroendocrinology*.

[12] Gileta A. F., Helgeson M. L., Leonard J. M. M. (2021). Further delineation of a recognizable type of syndromic short stature caused by biallelic SEMA3A loss‐of‐function variants. *American Journal of Medical Genetics, Part A*.

[13] Yoshida Y. (2012). Semaphorin signaling in vertebrate neural circuit assembly. *Frontiers in Molecular Neuroscience*.

[14] Giacobini P., Messina A., Morello F. (2008). Semaphorin 4D regulates gonadotropin hormone-releasing hormone-1 neuronal migration through PlexinB1-Met complex. *Journal of Cell Biology*.

[15] Laitinen E.-M., Vaaralahti K., Tommiska J. (2011). Incidence, phenotypic features and molecular genetics of Kallmann syndrome in Finland. *Orphanet Journal of Rare Diseases*.

[16] Alkelai A., Olender T., Dode C. (2017). Next-generation sequencing of patients with congenital anosmia. *European Journal of Human Genetics*.

