# Supplementary material for: Discovery of a Novel Variant of SEMA3A in a Chinese Patient with Isolated Hypogonadotropic Hypogonadism
Source: Int J Endocrinol. 2021 Oct 21;2021:7752526. doi: 10.1155/2021/7752526 (PMC8553509; doi:10.1155/2021/7752526)
Supplement: Supplementary Materials — Table S1: the sequences for PCR primers. [file 7752526.f1.docx]

Table S1 The sequences for PCR primers

| primer | Primer sequence |
| --- | --- |
| SEMA3A-F | 5’-CGAATTCATGGGCTGGTTAACTAGGATTG-3’ |
|  |  |
| SEMA3A-R | 5’-GGGATCCTCACTTATCGTCGTCATCCTTGTAATCGACACTCCTGGGTGCCCTCTCAAATTCGTG-3’ |
